# Iron and Folic Acid Supplementation Compliance and Associated Factors among Pregnant Women Attending Antenatal Clinic in Shalla District, Southwest Ethiopia: A Cross-Sectional Study

**DOI:** 10.1155/2021/6655027

**Published:** 2021-03-27

**Authors:** Aman Kedir Obsa, Yadesse Tegene, Achamyelesh Gebretsadik

**Affiliations:** ^1^Shalla District Health Office, Oromia, Ethiopia; ^2^Hawassa University College of Medicine and Health Science, Hawassa, Ethiopia

## Abstract

**Background:**

Iron-folate supplementation for a pregnant mother is a cost-effective intervention to reduce iron deficiency anemia during pregnancy. The aim of this study was to assess the iron-folic acid supplements and associated factors among pregnant women attending antenatal clinics in the public health center of Shalla district, Southwest Ethiopia.

**Methods:**

Institutional-based cross-sectional study design was conducted among 402 randomly selected pregnant mothers between February and April 2019. Data were collected using an interviewer-administered structured questionnaire from pregnant mothers attending antenatal care and using iron-folate supplements. Descriptive and multivariate logistic regression analyses were employed.

**Results:**

Pill count compliance rate was found to be (154) 38.3%. Pregnant mothers who had anemia in their previous pregnancy [(AOR = 11.35, 95% CI: 4.76–27.03)], counseling on iron-folate supplements [(AOR = 11.39, 95% CI: 5.09–27.03)], awareness of the benefit of the iron-folate supplements [(AOR = 2.22, 95% CI: 1.18–3.92)], and being a member of the Health Development Army [(AOR = 2.11, 95% CI: (1.2, 3.9)] were significantly associated with compliance with iron-folate supplement.

**Conclusion:**

Compared to the World Health Organization cut-off point, the pill count compliance rate of iron-folate supplementation among pregnant women in the study area was very low. Previous history of anemia and lack of knowledge about its benefit were some of the factors associated with it. Therefore, the healthcare providers should give continuous awareness creation and counseling services focusing on the benefit of iron-folate supplementation for pregnant mothers and their neonates.

## 1. Introduction

Iron and folic acid supplementation for pregnant mothers are the main strategies to prevent anemia among pregnant mothers and newborns even though all the above intervention works depend on the context of the causes of anemia [1–3]. The World Health Organization has recommended a six-month regimen of a daily supplement containing 30–60 mg of elemental iron with 400 *μ*g folic acid which is supplied to pregnant mothers during the first trimester or as soon as possible later on and it is provided when pregnant mothers come for antenatal care [3, 4]. In areas where the prevalence of anemia in pregnant mothers is >40%, anemia is a severe public health problem. Iron and folic acid supplementation should continue for three months in the postpartum period. Accordingly, in Ethiopia, the national guideline for control and prevention of micronutrient deficiency also recommends daily iron supplementation for 180 days during pregnancy or it could be finished after delivery if the mother did not finish the full dose during pregnancy [3, 5].

Compliance with the medication regimen is generally defined as the extent to which a patient takes medication as prescribed by their healthcare providers [6]. Increasing compliance with the medication has a greater effect on health than improvement in a specific therapy. On the contrary, noncompliance which is not taking medication as prescribed by a healthcare provider is the main obstacle to effective medical treatment. According to the World Health Organization report, noncompliance with any medication treatment varies from 15% to 93%, with an average estimated rate of 50% [6].

Ethiopian Demographic Health Survey (EDHS 2011) showed that, from all pregnant mothers supplemented with iron and folic acid tablet, only 0.4% consumed it for >90 days during their pregnancy time [7]. The EDHS 2016 finding also revealed that only 5% of pregnant mothers who gave birth five years before the survey took the supplements for the recommended period of time [8]. Therefore, the aim of this study was to assess the iron-folic acid supplements and associated factors among pregnant women attending antenatal clinics in the public health centers of Shalla district, Southwest Ethiopia.

## 2. Materials and Methods

### 2.1. Study Design, Period, and Setting

An institutional-based cross-sectional study was conducted in Shalla district, West Arsi Zone, which is found in the Oromia region of Ethiopia between February and April 2019. The district is one of the 20 districts in West Arsi Administrative Zone of Oromia located 280 km from the capital city, Addis Ababa, Ethiopia. According to projections based on the 2018 central statistical authority (CSA) population census of Ethiopia and district Health office data, the estimated total population for the year 2018 was 201,727 (8% urban and 92% rural), of whom 7,008 were pregnant women. The health service coverage of the district was 95%. In the district, there are 8 health centers, 38 health posts, and 5 private clinics [9]. Seven of the health centers, which are found in the district, provide free of charge regular antenatal care follow-up and iron-folate supplementation for the period of six months during pregnancy and the postpartum period.

### 2.2. Study Population

Pregnant women attending ANC clinics in selected public health centers of Shalla district during the data collection period who had at least one ANC visit in health centers, supplemented with IFA tablets for at least one month before the date of the interview and brought back a bottle of pills, were included in the study. Pregnant women with a serious illness were excluded from the study.

### 2.3. Sample Size and Sampling Technique

The sample size for compliance with iron-folate supplement and associated factors was calculated by using the formula to estimate a single population proportions *n*=(*Z* · *α*/2)^2^*p*(1 − *p*)/*d*^2^, *n* = sample size, *Z*·*α*/2 = significance level at *α* = 0.05, *P* = established prevalence from a previous study of the topic of interest (compliance) determinant of adherence to iron-folic acid supplementation among pregnant women attending antenatal clinic at Asella town (*p* = 59.8%) [10], and *d* = the margin of error of 0.05, with the assumptions of 95% confidence interval; using the above single population proportion formula, the sample size was calculated as *n* = (1.96)^2^0.392(0.608)/(0.05)^2^ = 366 and; considering a 10% nonrespondent rate, the total sample size was 402. The total estimated number of pregnant women attending antenatal clinics in each health center was taken depending on the health center monthly plan, assuming that antenatal care attendants have a similar flow. The sample size was proportionally assigned to each health center. The total sample size of 402 pregnant women who took ferrous-folate supplements during the previous appointments was selected using systematic random sampling from the source population of 814 in the district during 32 days of data collection.

### 2.4. Dependent Variable

Dependent Variable was compliant with the IFA supplement.

### 2.5. Independent Variables

  Sociodemographic: age, marital status, household monthly income, family size, place of residence, maternal educational level, husband's education, and occupation
  Obstetric factors: number of gravidae, parity, and number of ANC visits, abortion, previous history of anemia and ANC follow-up started time, and gestational age in months
  Women awareness: awareness of anemia, awareness about the benefits of IFA, and awareness about the duration of the supplementation
  Physical attributes and related factors: the side effects, forget fullness, unpleasant test, fear of big weight baby, and too many tabs harm infants
  Healthcare system: problem faced in the facility, shortage of supplements in the facility, and health education
  Health Development Army: organized groups of families who promote healthy activities and behavior among other families

### 2.6. Data Collection Methods and Procedures

The data collection instrument included six sections which are demographic, obstetrics history, knowledge on anemia and folic acid, compliance with iron and folic acid, and healthcare system factors. The questionnaire was developed by reviewing different literature and data were collected with a structured pretested questionnaire using an interview. The questionnaire was prepared in English and translated into Afaan Oromo and then back to English and compared for consistency. Data were collected by trained diploma midwives and interviewed pregnant mothers during antenatal care after getting consent.

### 2.7. Data Analysis

Data were entered using EpiData version 3.1 and analyzed using SPSS version 20. Compliance with the IFA supplement was assessed based on the pill count and self-report method. In the pill count method, mothers were given iron-folate for a period of one month and requested to bring back the bottle/container of the pill on the next appointment. Then, the bottle was checked for left pills in the bottle; if present, the number of pills in the bottle was counted, registered on a questionnaire, and considered as a number of pills expected to be taken/swallowed but not taken. In the self-report method, each pregnant woman was also assessed for the number of pills taken during the previous week before the interview. In both cases, compliance was defined as compliant if they took 70% or more of the prescribed supplements, which is equivalent to taking the supplements at least five days per week. The pill count method is more accurate than the self-reported method because it decreases the problem of recall bias [6, 11]. But both methods of compliance measurement have an additive effect if used together. The one-month period was selected based on the duration of iron-folate given in the Shalla District health facility:

(1) $$\begin{array}{c} \text{pill count compliance }\left(9\right)=\frac{\text{number of tablets taken in last month}\times 100}{\text{number of tablets prescribed in last month}}. \end{array}$$

Then, binary logistic regression was used to examine the relationship between the proposed predictors and compliance with iron-folate supplements. variable with *p* value ≤ 0.25 in the bivariate logistic regression analyses were considered as potential candidates in the final multivariable logistic regression analysis.

### 2.8. Operational Definitions

  Pregnant mothers' awareness about anemia: It was assessed using 15 questions. Respondents who scored above the mean were recorded as having good awareness of anemia.
  Pregnant mothers' awareness about the benefits of IFA supplement: It was assessed using three questions. Those who correctly answered above the mean were recorded as having good awareness of the benefit of iron-folate supplements.

## 3. Result

A total of 402 pregnant mothers attending antenatal care and taking iron-folate supplements in the Shalla district were included in the study, yielding a response rate of 99%. The mean age of the study participants was 25.21 (±SD 5.96) years. Most of the study participants, 285 (71%), were from a rural area, married 395 (98.3%), and attending secondary school (27%) (Table 1).

### 3.1. Past and Current Obstetrics Related Conditions

The gestational age of most of the study participants, pregnant mothers, ranges from 16 to 24 weeks; 226 (56%) were on the third ANC visit, 153 (38%) were multigravida, and 304 (76%) had no previous history of abortion and stillbirth 363 (90%). Majority of the study participants, 356 (89%), never heard about the disease anemia but had no history of anemia in both the current and past delivery, 384 (95.5%) and 249 (62%), respectively (Table 2).

### 3.2. Comprehensive Awareness of Benefit of Iron-Folate Supplement and Anemia

The comprehensive awareness of the benefit of iron-folate was assessed using three questions; those who correctly answered above the mean were recorded as having good awareness. One hundred twelve (28%) of the respondents had good awareness of the benefit of iron-folate supplements.

**Figure 1:**
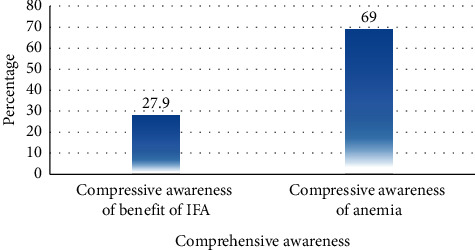
Compressive awareness of the benefit of IFA and anemia, among pregnant women attending antenatal care and taking iron-folate, Shalla district health facilities, West Arsi Zone, Southwest Ethiopia, 2019.

Compressive awareness of anemia was assessed using 15 questions: two questions on the symptom, four questions on the cause, four questions on the consequences, two questions on the susceptible group, and three questions on the prevention method; and each correct answer was given one point while wrong answers were given no points. Respondents scoring above the mean were recorded as having good awareness of anemia. Out of 356 respondents, 244 (69%) had good awareness of anemia (Figure 1).

### 3.3. Supplemental Related Factors

The majority, 376 (95%), of the interviewed pregnant mothers were taking their iron-folate supplements on a daily basis whereas 21 (5.2%) took the supplements on a weekly basis and, and about 5 (1.2%) took the supplements when they were sick (Table 3).

### 3.4. Health-Care-System-Related Factors

**Figure 2:**
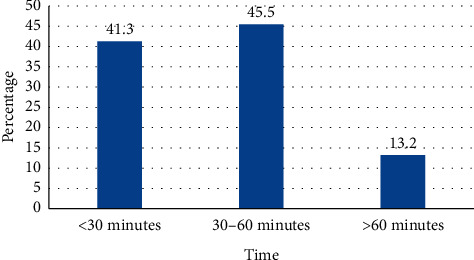
Time to reach the nearest health facilities from home among pregnant women attending antenatal care, Shalla district health facilities, West Arsi Zone, Southwest Ethiopia, 2011 (*N* = 402).

The majority of respondents, 183 (45.4%), reported that it takes thirty minutes to one hour to reach the nearest health facility and 164 (41%) respondents reported that it takes less than thirty minutes to reach the nearest health facility (Figure 2).

### 3.5. Compliance with Iron-Folate Supplement

**Figure 3:**
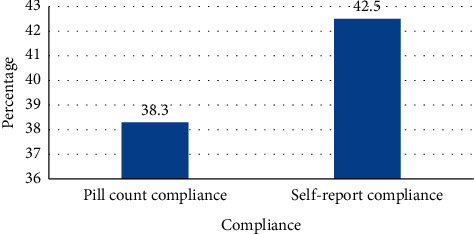
Compliance level with iron-folate supplement among pregnant women attending antenatal care and taking iron-folate, Shalla district health facilities, West Arsi Zone, Southwest Ethiopia, 2019 (*N* = 402).

Out of the total study participants, 154 (38.3%) (95% CI, 33.5%–43.1%) and 171 (42.5 %) (95% CI, 37.7%–47.4%) respondents were compliant according to the pill count and self-report to iron-folate supplement, respectively (Figure 3).

### 3.6. Factors Associated with Compliance

In the bivariate logistic regression analysis, anemia in a previous pregnancy, counseling service given on the importance of IFA, awareness of the benefit of IFA, being a health development member in Kebele, and a previous history of delivery were significantly associated with compliance with iron-folate supplement at *p* ≤ 0.25 and hence were used in the multivariate analysis.

Multiple logistic regression analyses revealing anemia in the previous pregnancy, counseling service given on the importance of IFA, awareness of the benefit of IFA, and being a health development member remain to be significantly associated with compliance with iron-folate supplement. Pregnant mothers who had a history of anemia during their previous pregnancy were 6 times [(AOR = 6.13, 95% CI: (2.9, 12.9)] more likely to be compliant than pregnant mothers who had no history of anemia in their previous pregnancy. Pregnant mothers who had received counseling service given on iron-folate supplement were 8 times [(AOR = 8.4, 95% CI: (4.5, 15.5)] more likely to be compliant than those who did not receive counseling service. Study participants who had awareness of the benefit of iron-folate supplements nearly 2 times [(AOR = 1.69, 95% CI: (1.0, 2.9)] were more likely to be compliant than those who did not have awareness of the benefit of iron-folate supplements. Those study participants who were members of the Health Development Army were also found to have two times more significant association [(AOR = 2.11, 95% CI: (1.2, 3.9)] than their counterparts (Table 4).

## 4. Discussion

In our study, 42.5% and 38.3 % of the respondents were compliant with iron-folate supplements according to the self-report and pill count methods, respectively. The finding of the level of pill count compliance is consistent with a study conducted in Pakistan, which is 38.35 [12], but lower than the study conducted in Addis Ababa, Akaki Kality (61%), and Egypt (48.9%) [12, 13], and higher than the study conducted in Uganda (11.6%) [14]. The probable reason for the difference could be attributed to the difference in sociodemographic variation. Compared to the World Health Organization cut-off point, compliance with iron-folate supplementation among pregnant women in the study area was low [6]. This could be due to supplemental factors associated with the drug, which make pregnant mothers skip their doses.

Pregnant mothers who had anemia in their previous pregnancies were more compliant than their counterparts. The finding is consistent with a study conducted in the Tigray region and the Mecha District of the Amara region [14, 15]. This could be due to the fact that experiencing symptoms of anemia in their previous pregnancy gave them awareness about the disease and to seek treatment in the present pregnancy.

Pregnant mothers, who got counseling service during their ANC visit on the benefit of iron-folate supplement, had higher odds of compliance than those who did not receive counseling service. This finding is in line with the study finding from Misha district, south Ethiopia, and the studies done in Kenya, India (Haryana state), Sweden, Cambodia, and Senegal [1, 16, 17].

Having awareness of the benefit of iron-folate supplementation was also found to have an association with compliance with iron-folate supplementation. Study participants who had awareness of the benefit of iron-folate supplements had higher odds of compliance than their counterparts. A similar finding was obtained from the study conducted in Senegal and the Mecha district of the Amara region. [13, 14].

The other factor which has shown significant association with compliance with iron-folate supplementation was being a member of a Health Development Army. Those study participants who were members of the Health Development Army were two times more likely to be compliant with iron-folate supplementation than those who were not. Though there are no study reports which indicate an association between compliance with iron-folate supplementation and being a Health Development Army, there are studies which indicate the existence of a strong association between pregnant mothers with good knowledge about iron-folic acid supplement and compliance with iron-folate supplements during pregnancy [15, 18]. The reason could be that knowledge helps women to have a good perception of iron-folic acid supplements.

Eighty-five percent of pregnant women who missed taking their supplementation were due to forgetfulness. This finding was higher than studies done on the Misha district of South Ethiopia (42.8%) and India (48.8%) [1, 19]. This difference may be due to the fact that the present study considers multiple responses, but the previous study allowed only one reason. In addition to this, provider differences in the geographic area and socioeconomic status of study participants may be the cause for the difference.

Even though the pill count compliance method was used to measure compliance, still there could be social desirability and recall bias, under/overestimated compliance rate. Generally, the gold standard measure of compliance is the electronic method, though it is difficult to implement in our setup because of its expensiveness.

## 5. Conclusion

In conclusion, pill count compliance rate, as well as self-report compliance with iron-folate supplementation among pregnant women in the study area, was low compared to the World Health Organization cut-off point. Anemia in a previous pregnancy, counseling service given on IFA, awareness of the benefit of IFA, and being a member of the Health Development Army remain to be significantly associated with compliance with iron-folate supplement. In addition, forgetfulness and fear of side effects of the iron-folate supplements in the health facility were commonly mentioned reasons for missing the doses of the iron-folate supplements. Therefore, the healthcare providers should provide continuous awareness creation and counseling services focusing on the benefit of iron-folate for pregnant mothers and their neonates. In addition, household members should encourage and remind pregnant women to take their supplements on a daily basis.

## Figures and Tables

**Table 1:** Sociodemographic characteristics of pregnant women attending antenatal care and taking iron-folate, Shalla district health facilities, West Arsi Zone, Southwest Ethiopia, 2019 (*N* = 402).

Variables	Frequency	Percent
Age		
15–20	85	21
21–25	137	34
26–30	83	21
31–39	97	24

Residence		
Rural	285	71
Urban	117	29

Marital status		
Married	395	98
Widowed	4	1
Separated	3	1

Educational status of the mother		
Cannot write and read	85	21
Can write and read	129	32
Primary school 1–8	82	20
Secondary school 9–10	74	18
Above secondary	32	8

Educational status of partner		
Cannot write and read	54	13
Can write and read	67	17
Primary school 1–8	82	20
Secondary school 9–10	107	27
Above secondary	92	23

**Table 2:** Past and current obstetric history of pregnant women attending antenatal care and taking iron-folate, at Shalla district health facilities, West Arsi Zone, Southwest Ethiopia, 2019 (*N* = 402).

Variables	Frequency	Percent
Gestational age at first antenatal visit		
Less than 16 weeks	78	19
16–24 weeks	226	56
25 and above weeks	98	24

Number of antenatal visits		
Second visit	123	31
Third visit	153	38
Fourth visit	83	21
Above fourth	43	11

Previous history of delivery		
Yes	304	76
No	98	24

Previous history of abortion		
Yes	42	10
No	360	90

Previous history of stillbirth		
Yes	39	10
No	363	90

Ever heard of anemia disease		
Yes	356	89
No	46	11

Anemia in the current pregnancy		
Yes	18	4.5
No	384	95.5

Anemia in a previous pregnancy (*N* = 304)		
Yes	55	38
No	249	62

Being Health Development Army		
Yes	226	56
No	176	44

**Table 3:** Reason for noncompliance with iron-folate supplements among pregnant women attending antenatal care and taking iron-folate, Shalla district health facilities, West Arsi Zone, Southwest Ethiopia, 2019 (*N* = 402).

Variables	Frequency	Percent
Reason for noncompliance with IFA		
Forgetfulness	313	85.1
Fear of pill side effect	218	59.2
Dislike test pill	28	7.6
Too many pills	2	0.5

Side effects preventing taking IFA		
Epi gastric burning pain	137	64.0
Vomiting	114	53.3
Constipation	15	7.0
Diarrhea	3	1.4

**Table 4:** Multivariate logistic analysis of factors influencing compliance with iron-folate supplement among pregnant women attending ANC in Shalla district health facilities, West Arsi Zone, Ethiopia, 2019 (*N* = 402).

Variables	Compliance with iron-folate supplement		COR (95% CI)	AOR (95% CI)
	Compliant *N* (%)	Noncompliant *N* (%)		
Anemia in a previous pregnancy (*N* = 307)				
No^®^	160 (87.4)	90 (72.6)	2.63 (1.5–4.7)	**6.13 (2.9, 12.9)**
Yes	23 (12.6)	34 (27.4)	1	1

Counseling service given on IFA				
No^®^	98 (39.5)	121 (78.6)	1	1
Yes	150 (60.5)	33 (21.4)	5.61 (3.6, 8.9)	**8.4 (4.5, 15.5)**

Awareness of the benefit of IFA				
No^®^	188 (75.8)	102 (66.2)	1	1
Yes	60 (24.2)	52 (33.8)	1.59 (1.1, 2.49)	**1.69 (1.1–2.9)**

Member of Health Development Army				
No^®^	133 (53.6%)	133 (53.6%)	1	
Yes	43 (27.9%)	43 (27.9%)	2.99 (1.9, 4.6)	**2.11 (1.2, 3.9)**

Previous history of delivery				
No^®^	67 (27.0%)	31 (20.1%)	1	
Yes	181 (73.0%)	123 (79.9%)	1.5 (0.9, 2.4)	1.07 (0.1, 15.4)

Statistically significant variables in multiple logistic regressions at *p* value ≤0.05. ® Reference category. IFA, iron and folic acid; COR, crud odds ratio; AOR, adjusted odds ratio; CI, confidence interval.

## Data Availability

The datasets used and/or analyzed during the current study are available from the corresponding author on reasonable request.

## Abbreviations

ANC: Antenatal care
CSA: Central Statistics Agency
DHS: Demographic Health Survey
EDHS: Ethiopian Demographic Health Survey
FA: Folic acid
HC: Health center
HDA: Health Development Army
Hgb: Hemoglobin
ID: Iron deficiency
IFA: Iron and folic acid
PW: Pregnant mother
SPSS: Statistical Package for Social Science
SRS: Simple random sampling
WHO: World Health Organization.
